# Evaluation of an oral health education training program for kindergarten teachers

**DOI:** 10.3389/froh.2024.1503221

**Published:** 2025-01-15

**Authors:** Joanna Cheuk Yan Hui, Chun Hung Chu, Jieyi Chen

**Affiliations:** ^1^ Hospital of Stomatology, Sun Yat-sen University, Guangzhou, China; ^2^ Faculty of Dentistry, The University of Hong Kong, Hong Kong, Hong Kong SAR, China

**Keywords:** oral health, early childhood caries, health education, preventive dentistry, public health

## Abstract

**Objectives:**

This study aims to examine kindergarten teachers’ satisfaction and their perceived impact of an oral health education (OHE) training program.

**Methods:**

This study followed the steps outlined in the Program Evaluation Standards in Public Health recommended by the Centers for Disease Control and Prevention. All kindergarten teachers in Hong Kong were invited to attend the OHE training. The training comprised a lecture and a group discussion among six to eight teachers facilitated by trained dentists. The participating teachers were invited to complete an online questionnaire after the training. The questionnaire consisted of seven questions: six close-ended questions to evaluate teachers’ satisfaction with the training, their perceived oral-health-related knowledge, and their perceived competence in delivering OHE, and one open-ended question to collect their comments and suggestions for enhancing the OHE training.

**Results:**

A total of 6,210 teachers joined the OHE training and 4,882 teachers completed the questionnaires (response rate: 79%). The survey found that 4,691 (96%) teachers were satisfied with the training, and the same number agreed that the OHE training was essential for delivering OHE. In addition, 4,680 (96%) teachers found the lecture informative and 4,393 (90%) teachers rated the group discussion as useful. After the OHE training, 4,716 (96%) teachers reported that they were more knowledgeable in recognizing childhood oral diseases and 4,665 (96%) teachers believed they were more skillful in delivering OHE. Regarding comments and suggestions, teachers opined that the OHE was informative and comprehensive. They suggested developing videos and hands-on workshops for the OHE training and expressed a desire to learn more about the dental treatment of common dental diseases. They also recommended providing teaching aids, such as booklets and tooth models, for use in kindergartens.

**Conclusion:**

The kindergarten teachers generally had a better understanding of childhood oral conditions after the OHE training. They were generally satisfied with the training and became more confident in delivering OHE to kindergarten children.

## 1 Introduction

Dental caries or early childhood caries (ECC) is a childhood common chronic infectious disease (1). The disease of early childhood caries is defined as one or more decayed, missing (resulting from caries), or filled teeth in primary dentition in children aged up to 71 months (2, 3). ECC remains prevalent worldwide, with over 573 million children experiencing dental caries (4). In Hong Kong, over 50% of 5-year-old children experience tooth decay or ECC, with more than 90% of their carious lesions remaining untreated (5). The impact of untreated caries in early childhood is not just pain and infection but more immediate and long-term consequences (6). ECC is a health burden to the family and society, including higher risk of acute and chronic pain, hospitalizations and emergency room visits, delays in growth and development, and diminished quality of life, all of which are costly for society, government, and family (7, 8). Given the high prevalence of untreated ECC reported in Hong Kong and globally, there is an urgent need to prevent this disease (9).

Over the years, several preventive programs have been implemented by the Hong Kong Government to reduce dental caries in children. The School Dental Care Service was launched in the 1980s to provide regular check-ups and treatments for primary school students (10). However, preschool children are not eligible to join this service. Oral health education (OHE) programs, such as Brighter Smiles Play Land, are offered to preschool children by the Oral Health Education Division under the Department of Health. Nevertheless, no preventive or curative treatment is available for preschool children from these programs (11). To date, there is no primary oral health program targeting ECC at the government level. To improve the ECC situation, a comprehensive oral health program called Children Oral Health Program was launched by The University of Hong Kong to provide dental care services to all preschool children in Hong Kong (approximately 160,000 children). This program was funded by a charity organization to provide dental services from 2019 to 2022 initially, and then received extra funding to provide services from 2023 to 2026.

The program offers a comprehensive range of services, including on-site dental check-ups and silver diamine fluoride treatment, individual consultation and oral health education for parents, and oral health education training for teachers. The World Health Organization (WHO) suggests that engaging different stakeholders, including teachers and parents, is of vital importance to manage ECC (12). The online OHE training for teachers in this program consisted of a 90-min lecture followed by a 60-min small group discussion modulated by four trained dentists. Between 2019 and 2022, a total of 68 training sessions were conducted fortnightly, excluding public holidays. The lecture content covered essential topics, including oral health status among preschool children, etiology and risk factors of dental caries, prevention and treatment options for dental caries, other common oral health problems and treatments, and the teacher's role and teaching technique suggestions. In small group discussions, the dentists demonstrated tooth-brushing and flossing techniques, examined the acidity of common beverages, answered questions, and invited teachers to share their experiences and difficulties in providing OHE in preschools. The teachers were encouraged to practice tooth brushing and flossing as well as testing common beverage acidity by themselves. The details about the training curriculum can be found in Supplementary File 1. All kindergarten teachers in Hong Kong were invited to attend this optional training. The aim of the OHE training is to empower teachers with knowledge to recognize childhood oral diseases, enhance their skills in delivering oral health education, and ultimately improve children's oral health.

Numerous studies have reported that the prevalence of ECC is significantly related to socioeconomic factors as well as behavioral factors, such as oral hygiene and dietary practices (13, 14). Teachers are one of the key individuals in reinforcing the oral health message and promoting oral-health-related habits to children. Preschool teachers, namely, early childhood educators, are pivotal in shaping the development paths of young children (15, 16). There are strong, responsive relationships between child and caregiver, including early childhood educators, in facilitating children's communication and health behavior development (17, 18). Teachers contribute to children's educational development and influence their wellbeing with daily routines, including essential oral hygiene habits. They interact with children frequently and act as primary influencers of children's daily behaviors (19). Moreover, they usually act as the primary contact point for parents regarding their child's development and needs, and as an influencer to both child and family health-related practices (20, 21). Equipping them with proper training in oral health has substantial potential in enhancing their ability in promoting good oral hygiene habits among children (18).

It is important to understand the impact of OHE training on kindergarten teachers. We can identify gaps to enhance the training and provide evidence-based updates for the dental professionals and policymakers to plan long-term policy and formulate a comprehensive health promotion strategy. Our experience can be contributed to other school-based health programs worldwide. This study aims to examine kindergarten teachers’ satisfaction with the OHE training and their perceived impact of the training. In addition, the study also collected their comments and suggestions toward the OHE training.

## 2 Materials and methods

### 2.1 Ethical consideration and study design

**Figure 1:**
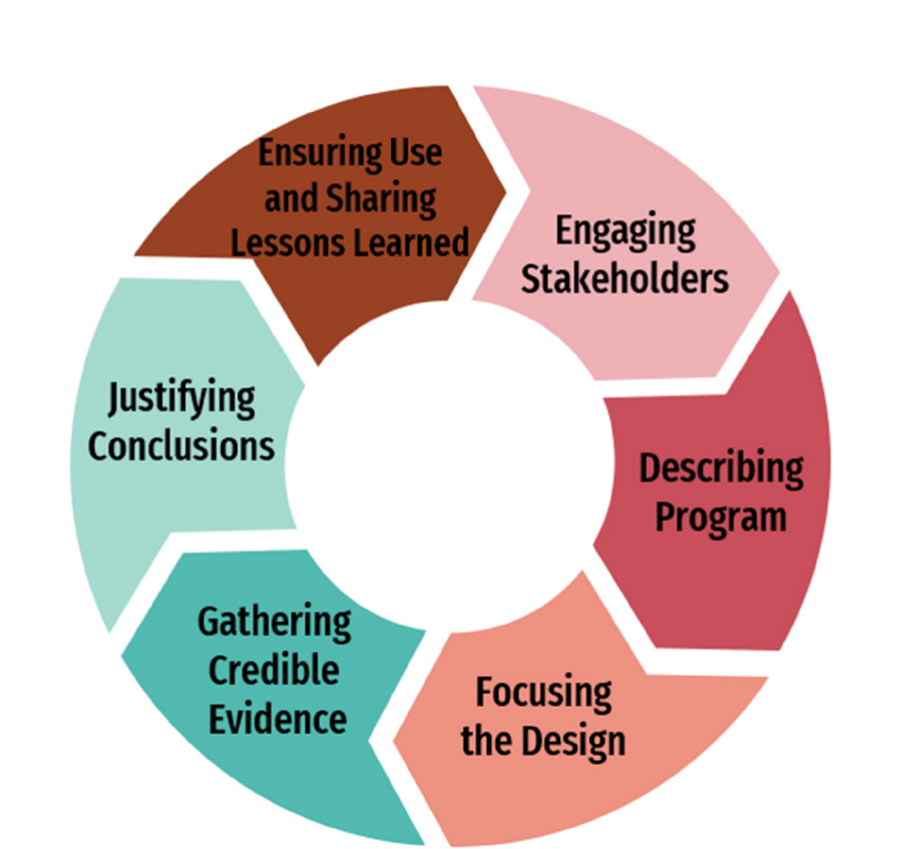
CDC framework for program evaluation in public health.

This study obtained ethical approval from the Institutional Review Board of the University of Hong Kong Hospital Authority Hong Kong west cluster (UW 19-660). The study collected anonymous responses for confidentiality. Participants were fully informed about the purpose of the study, the nature of their involvement, and the data handling procedures. They could withdraw from the survey at any time. This study is a program evaluation guided by the Program Evaluation in Public Health framework from the Centers for Disease Control and Prevention (CDC), consisting of six interconnected, non-linear steps including (1) engaging stakeholders, (2) describing the program, (3) focusing the design, (4) gathering credible evidence, (5) justifying conclusions, and (6) ensuring use and sharing lessons learned (22) (Figure 1).

### 2.2 Participants

This territory-wide children's oral health program aimed to cover all kindergartens in Hong Kong. At the initial stage (2019–2022), over 70% of kindergartens participated. All teachers at the participating kindergartens were invited to join the training, which was organized as part of their continuous education and held at the kindergartens.

### 2.3 Data collection and method

The questionnaire included close-ended and open-ended questions. The online questionnaire was distributed after the training and data were collected between January 2020 and December 2022. The questionnaire was written in Chinese and English (the Chinese version shall prevail) to gather information regarding participants’ satisfaction toward the training, perceived oral-health-related knowledge, and competence in delivering OHE, as well as their comments and suggestions to enhance the training. The closed-end questions used a 5-point Likert scale, a commonly used psychometric tool, ranging from 1 (strongly agree) to 5 (strongly disagree). An open-ended question was designed to capture participants’ insights into the OHE training (Table 1). Participants were encouraged to review their responses for completeness and accuracy before submission to eliminate the possibility of missing, unclear, or inappropriate responses, ensuring the integrity of the data collected.

**Table 1:** The themes and questions in the evaluation questionnaire.

Section	Themes	Questions
1. Close-ended questions	1. Teacher training essentiality	Do you agree the teacher training is essential for kindergarten teachers?
	2. Lecture informativeness	Do you agree the Lecture provided is informative?
	3. Group discussion usefulness	Do you agree the group discussion is useful?
	4. Knowledge enhancement in recognize pediatric oral diseases	Do you agree you become more knowledgeable in recognizing pediatric oral diseases after the teacher training?
	5. Skills enhancement on delivering oral health education for kindergarten	Do you agree you become more skillful in providing oral health education to kindergarten children after the teacher training?
	6. General satisfaction	Do you generally satisfy with the teaching training?
2. Open-ended question	7. Perspective and Suggestions for the training	Please provide your comments suggestions for the teacher training.

### 2.4 Data analysis

The quantitative data from close-ended questions was analyzed using SPSS version 28 (IBM Corp., Armonk, NY, USA). Descriptive statistics were employed to report the data. Qualitative contents from open-ended question were manually coded and analyzed following the thematic analysis process proposed by Virginia Braun and Victoria Clarke (23).

## 3 Results

**Figure 2:**
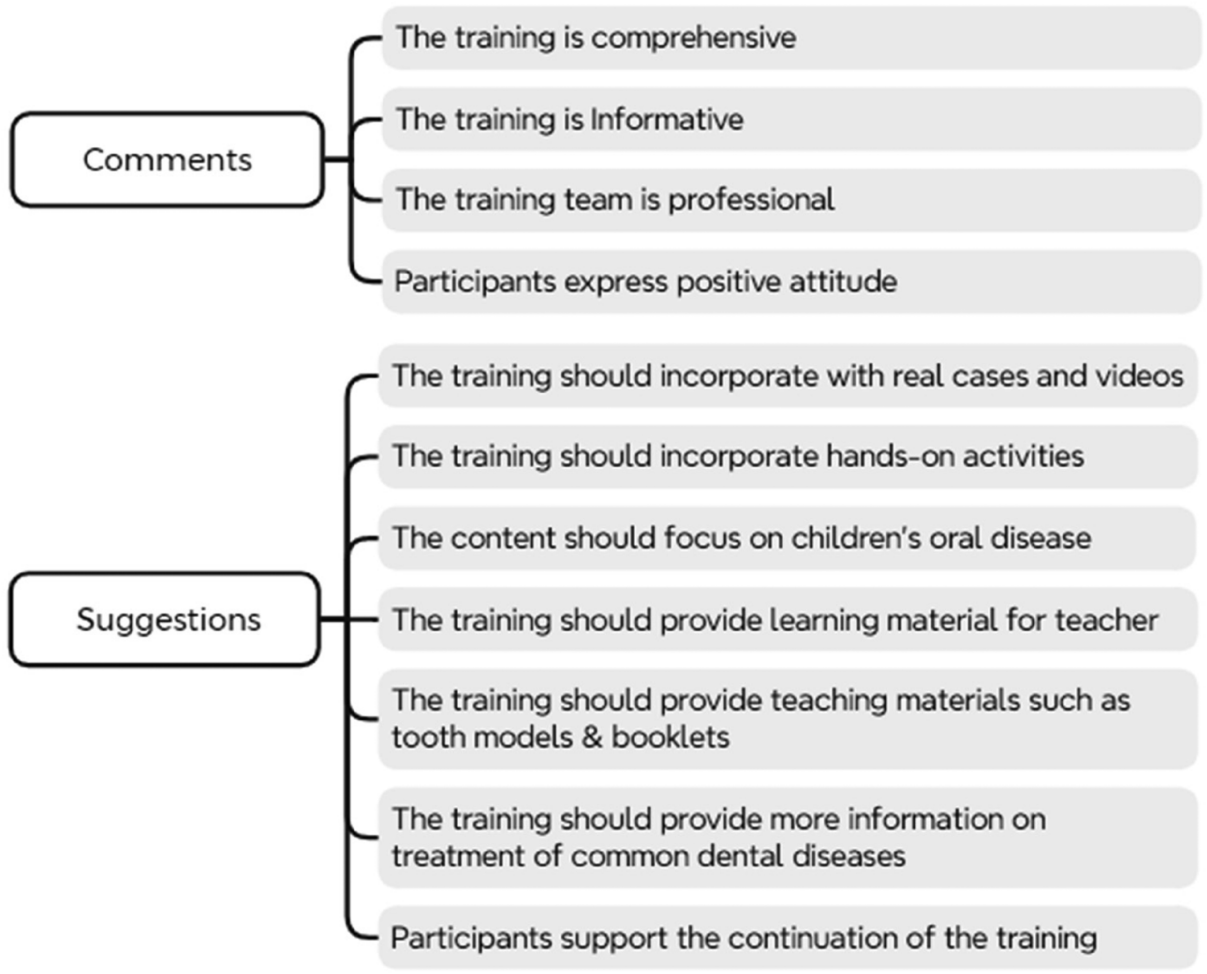
Thematic framework of teachers’ perspectives and suggestions on teacher training.

This study had 6,210 kindergarten teachers participating in the training between 2019 and 2022. Of them, 4,882 completed the survey (response rate 78.6%). The findings are summarized below and presented in Table 2 and Figure 2.

**Table 2:** Teachers’ satisfaction and enhancement in their knowledge and skills after training (*N* = 4,882).

Questions	Strongly agree	Agree	Neural	Disagree	Strongly disagree
1. Do you agree the teacher training is essential for kindergarten teachers?	1,969 (40.3%)	2,722 (55.8%)	177 (3.6%)	8 (0.2%)	6 (0.1%)
2. Do you agree the lecture is informative?	1,951 (40%)	2,729 (55.9%)	193 (4%)	5 (0.1%)	4 (0.1%)
3. Do you agree the group discussion is useful?	1,537 (31.5%)	2,856 (58.5%)	465 (9.5%)	20 (0.4%)	4 (0.1%)
4. Do you agree you become more knowledgeable in recognizing childhood oral diseases after the teacher training?	1,973 (40.4%)	2,743 (56.2%)	156 (3.2%)	8 (0.2%)	2 (0.0%)
5. Do you agree you become more skillful in providing oral health education to kindergarten children after the teacher training?	1,811 (37.1%)	2,854 (58.5%)	204 (4.2%)	12 (0.2%)	1 (0.0%)
6. Do you generally satisfy with the teaching training?	1,810 (37.1%)	2,881 (59%)	181 (3.7%)	7 (0.1%)	3 (0.1%)

### 3.1 Teacher satisfaction

The results indicated that 96.1% of participants agreed or strongly agreed that the overall training was essential, 95.9% agreed or strongly agreed that the lecture was informative, and 90% agreed or strongly agreed that the group discussions were useful. The overall satisfaction of the teacher training was 96.1%.

### 3.2 Knowledge and skill enhancement

The results demonstrated that 96.6% of participants reported they were more knowledgeable in recognizing childhood oral diseases after the training and 95.6% indicated they were more skillful in delivering oral health education to children after the training.

### 3.3 Comments about the training

Participants commented that the training was comprehensive. The training effectively provided valuable information regarding children's oral health, furnishing their insights into the importance of oral health.

“Training information is comprehensive.”

“The content is rich, letting us know the precautions for oral development and the steps, methods and importance of dental care.”

The participants widely perceived the training as informative. They reported that the training had enriched their knowledge and understanding relevant to children’s oral health. Some participants commented that the training allowed them to gain more practical insights and knowledge of the precautions about daily beverages.

“The information provided was relevant and practical, greatly enriched the teachers’ knowledge.”

“I think this training can effectively help me understand the oral problems of young children.”

“The drinks test allows everyone to understand the pH of drinks we have daily and their influence, so that we will not choose the wrong drink.”

Participants appreciated the training team. They commented that the lecturer and staff were professional and able to answer different questions immediately.

“Thank you, the team to prepare the training, the arrangement is smooth and everyone can ask question immediately”

“Appreciation to the dentist and staffs in training. They have the passion on helping children improve oral health”

“The staffs can answer my questions professionally.”

The participants expressed a positive attitude toward the teacher training. They stated that the training provided positive engagement and interaction.

“Very good. Paying attention to the importance of dental cleansing for child.”

“Appreciation to the training. The training can increase our awareness of oral health care”

### 3.4 Suggestions for the training

Some participants suggested incorporating real-life cases or videos to provide more in-depth insights into dental treatments and their relevance to children's oral health.

“Better to share some real special cases.”

“Should provide videos with more in-depth treatment process about the tooth decay.”

“Share more cases from previous check-ups as examples.”

“Providing simulation videos to illustrate the progression of tooth decay and the repair process would be useful.”

Some participants suggested incorporating hands-on activities to enhance engagement and deepen their understanding of oral health topics. They emphasized the value of interactive learning experiences in training sessions.

“I appreciate experimental activities in training; these kinds of activities make me more memorable in what I learn.”

“Some interesting workshop activities or small experimental games can be held to enhance our understanding of oral health.”

“Do some hands-on activities with teachers.”

Participants advised the training should focus on preschool children and reduce the knowledge about adults. They also suggested more information should be added, including toothpaste and toothbrush selection as well as referral channels.

“Can reduce the adult part and focus on children.”

“Can only describe the dental situation of children aged 3 to 6 years old, omitting dental implants, dentures, etc.”

“Can provide more explanations on treatment or referral channels”

“Please recommend some toothpaste and toothbrushes suitable for children”

Participants mentioned the importance of accessibility to dental knowledge resources after the training. They would like to have convenient access to the lecture's PowerPoint, notes, and reading materials.

“It would be ideal if, after completing the training, a PowerPoint would be distributed via schools’ emails.”

“It would be better to provide notes, as the handwriting is slow.”

“It would be better if there were reading materials for teachers to archive and review the information”

Participants emphasized the necessity of providing educational resources. They believed these resources would help them engage students and deliver knowledge better. Some participants suggested providing booklets to enhance their understanding of oral health. They emphasized well-designed educational materials in promoting good dental practices.

“Better provide the teaching material to kindergarten.”

“Send us tooth model, so we can remind everyone of the common mistakes on brushing and flossing.”

“It would be good to send us tooth models to demonstrate the children on how to brush and floss your teeth.”

“It would be helpful to provide booklets for us and the children that highlight key points on how to keep teeth healthy.”

“Offering booklets or leaflets with storytelling and cartoons would make the information more engaging and memorable.”

Participants suggested that the training could provide more explanations regarding tooth replacement and the abnormalities of dental development. Furthermore, they recommended that the training could provide more in-depth information that addresses both the effects of unresolved serious dental problems and the management of dental emergencies.

“In addition to talking about tooth decay issues, I hope there will be an explanation about the tooth replacement. For example, is it normal for the teeth to become sparse before the replacement?”

“More in-depth information should be provided about the effects of unresolved serious dental problems.”

“It would be helpful to have information on the signs or symptoms that indicate a child may have an oral disease.”

“Tell us more about how to handle accidents like teeth trauma, would be very useful!”

Participants have expressed their willingness at continuous training. They suggested regular and annual training may improve teachers’ overall knowledge as there are many new teachers every year.

“The training should conduct regularly to teach the new kindergarten teachers every year.”

“Should keep holding the teacher training.”

## 4 Discussion

This is the first study to evaluate a program-based OHE training for kindergarten teachers in Hong Kong. This territory-wide school-based outreach oral health program is the first of its kind, an eye-catching program benefiting over 100,000 preschool children. Teacher training is an essential part of this program because effective collaboration and engagement with different stakeholders are crucial to the success of oral health promotion (20). The results of this study showed that the OHE training demonstrated a high level of engagement and satisfaction among participants. The training was essential for their teaching in kindergartens and participating teachers generally valued the training and its benefits. They reported that the training helps equip them with more knowledge to recognize children’s oral diseases and to deliver oral health education to children. The training met teachers’ expectations and was recommended to continue in the long term. Moreover, participants raised comments and suggested different key elements that may also act as references for other health programs. They commented that the training is comprehensive and informative, significantly enhancing their knowledge about children's oral health. They appreciated that the rich content covered essential precautions for dental caries and effective dental care methods. Moreover, the professionalism of the training team was highly praised. In general, this OHE training met its goal of empowering preschool teachers.

Participants offered valuable suggestions to enhance the training. They suggested the content of the training should be more realistic and closer resemble daily situations. Some studies suggested that case-based learning using real-life cases helps learners impart relevance and connect the theory to reality (24). In addition, interactive and hands-on activities should be increased because traditional lectures have certain limitations. Interactive tools help to attract people's attention and participation, which may also benefit the implementation of the training (25, 26). Moreover, more resources and support were also considered necessary for the training. People tend to seek more information and practical support after realizing the importance of oral health. Providing educational resources after training, such as PowerPoint documents, notes, and reading materials, are necessary to aid teachers in their ongoing learning and teaching efforts. Educational resources may help people learn about the related topic and post (27). Teaching materials, including tooth models and booklets, are useful tools to facilitate demonstrations of proper dental care techniques. This practical support may help to solve problems and be effective in transferring and applying the skills and behavior learned during training to the teacher's workplace (27, 28). Therefore, this program has undergone significant adjustments in phase II (2023–2026). The teacher training has been revamped to include more real-life cases and various interactive elements such as hand-on activities. The training has been refined, with more focus on children's oral health topics, and expanded to include more content on different treatment option, knowledge of tooth replacement, dental development abnormalities, and the management of dental emergencies. In addition, different teaching and learning materials, such as tooth models and booklets, are offered. The impact of the teacher training in phase II will be evaluated in a future assessment.

The WHO suggested that the school setting provided a unique platform for implementing population-directed oral health interventions (20). Children's participation was increased in a school setting and those who were unable to go to dental clinics could be reached (29). Moreover, school is the place where students develop lifelong health-related behaviors, beliefs, and attitudes, which may help develop good oral health awareness and behaviors (12, 26, 30). Professional training is a major component of updating teachers' knowledge and skills in different categories (31). A key step to establishing collaboration with schools is to provide effective professional preparation for childhood educators. A study conducted in Hong Kong kindergartens indicated that teachers’ understanding of a health program might affect their attitudes toward the implementation of the program in their kindergartens (32). A literature review showed that teacher advocacy for health programs can effectively influence their implementation (33). Therefore, it is important to involve teachers in school-based programs in advance, trigger their interest, and empower them to teach children about oral health in a school setting. Teacher training is crucial to the school-based health program's success and is beneficial to kindergarten children in improving their oral health. The WHO also suggests that both parents and teachers play a crucial role in children's oral health behaviors and attitudes. A literature review revealed that many parents are not adequately trained and informed about managing their children's oral hygiene. The gap in oral health literacy can lead to a range of negative oral health outcomes (34). Therefore, parents’ oral health education is also important (35). It is essential to develop and implement a comprehensive program promoting oral health at school to empower caregivers to manage oral-health-related risk factors among their children.

This study was conducted following the Program Evaluation Standard in Public Health framework from the CDC. To gather evidence from participants, this study adopted closed-ended questions to collect solid results as well as open-ended question to allow participants to express detailed insights and discover unanticipated responses (36, 37). To manage diverse responses from the open-ended questions, a thematic analysis was adopted to discover, interpret, and report patterns and clusters of meanings. Moreover, this study used themes and coding during data analysis to facilitate the process of summarizing key issues and to present the findings in a clear and compelling narrative (36).

However, this study has some limitations. First, the program conducted online OHE training during the COVID-19 epidemic during 2019–2022. This setting may influence participants’ experiences, potentially differing from face-to-face training. Second, participants in this optional training might be influenced by social desirability bias, leading to favorable and positive feedback. Third, the materials and curriculum are not universally recognized and may need to be validated or updated if they are adopted by other countries. Nevertheless, this study helps researchers, project managers, and different stakeholders understand teachers’ views toward school-based oral health education training for teachers and provides different ways of improving the framework, service, and lesson to plan future health programs.

## 5 Conclusion

The participating teachers were generally satisfied with the training and agreed that the training achieved its objective of equipping them with knowledge and skills. They expressed positive attitudes toward the training. They agreed that this training was beneficial and suggested that it was implemented regularly, providing suggestions for its content and resources. This study provided evidence on the impact of this program-based oral health education training for kindergarten teachers in Hong Kong.

## Data Availability

The raw data supporting the conclusions of this article will be made available by the authors, without undue reservation.
