# Supplementary material for: Evaluation of an oral health education training program for kindergarten teachers
Source: Front Oral Health. 2025 Jan 15;5:1503221. doi: 10.3389/froh.2024.1503221 (PMC11774879; doi:10.3389/froh.2024.1503221)
Supplement: Supplementary file 1 [file Datasheet1.pdf]

Supplementary File 1: Online OHE Training Curriculum for Teachers

Outline	Content	Methods	Time
	Lecture (1.5 hour)		
1. Introduction of oral health status among preschool children	• Oral health status among preschool children in Hong Kong<br>• Definition of dental caries and its influence on preschool children	PowerPoint presentation by 1 trained dentist	15 min
2. Etiology and risk factors of dental caries	• Anatomy of the tooth<br>• Etiology of dental caries and risk factors	PowerPoint presentation by 1 trained dentist	20 min
3. Prevention and treatment options for dental caries	• Preventive strategies for dental caries including the use of fluoride agents, toothbrushing, flossing and diet suggestions<br>• Dental caries treatment options	PowerPoint presentation by 1 trained dentist	20 min
4. Other common oral health problems and their treatment among preschool children	• Introduction of staining, dental trauma, periapical periodontitis, malocclusion, and permanent teeth eruption<br>• Treatment options for these oral health problems	PowerPoint presentation by 1 trained dentist	20 min
5. Teacher's role and teaching technique suggestions	• Suggested teaching strategies for providing OHE to preschool children<br>• Role of teacher and what they can suggest to parents/caregivers to promote children's oral health	PowerPoint presentation by 1 trained dentist	15 min
	Small Group Discussion (1 hour)		
6. Demonstration and practice for oral hygiene practice	• Demonstration of tooth brushing and flossing techniques with tooth model<br>• Practice tooth brushing and flossing by teachers at their place	Demonstration and videos by 4 trained dentists; Practice by teachers	15 min
7. Experiment on common drinks' pH values	• Introduce the pH values of different types of drinks<br>• Demonstrate the effects of drinks at different pH values on egg shells<br>• Teachers are encouraged to try to test the pH values of common drinks	Demonstration and videos by 4 trained dentists; Practice by teachers	15 min
8. Discussion, question and answer	• Discussion on the importance of oral health education for children<br>• Answering questions and providing clarifications of the training	Discussion modulated by 4 trained dentists	20 min
9. Experience Sharing	• Sharing experiences, difficulties and insights on implementing OHE in preschools by teachers	Sharing and discussion	5 min
10. Conclusion and Evaluation (5 minutes)	• Review of key points of the OHE training	PowerPoint presentation	5 min

- Before the training, the teachers should prepare toothbrush, dental floss and pH test strips by themselves.
